# Development and validation of a diagnostic test for Ridge allele copy number in Rhodesian Ridgeback dogs

**DOI:** 10.1186/s40575-015-0013-x

**Published:** 2015-01-27

**Authors:** Jennifer Turner Waldo, Kasandra Santana Diaz

**Affiliations:** Biology Department, State University of New York at New Paltz, 1 Hawk Drive, New Palz, NY 12561 USA

**Keywords:** Copy number variation, Genotyping, qPCR, Rhodesian Ridgeback, Dermoid sinus

## Abstract

**Background:**

The breed-defining dorsal ridge in Rhodesian Ridgeback dogs is the result of a 133,000 base pair duplication on chromosome 18. Because this trait is dominant, heterozygous dogs cannot be discriminated from those with two copies of the Ridge allele.

**Results:**

A quantitative PCR test was developed and dogs of known genotype were used as test subjects. In all cases, the correct genotype was determined experimentally.

**Conclusions:**

This work provides a rapid and accurate methodology for determining dog genotype with respect to the Ridge allele.

## Lay Summary

The breed-defining ridge on the back (dorsal ridge) of Rhodesian Ridgeback dogs is the due to the presence of a duplication of a specific region on chromosome 18, which has resulted in multiple gene copies.

This particular duplication includes the Ridge gene, and dogs having this duplication on one of their two chromosome* 18s will have three copies of the gene (1+2 not 1+1), while dogs with a duplication on both their chromosomes will have four copies (2+2 not 1+1). Dogs with no duplication will have two copies of the gene (1+1), but this results in no dorsal ridge. As the ridge trait is dominant, heterozygous dogs (with one duplication) cannot be discriminated from those with two duplications of the Ridge allele.

A DNA test has been developed and dogs of known genotype were used as test subjects. In all these test samples, the correct genotype was determined experimentally.

This work provides a rapid and accurate methodology for determining dog genotype with respect to the Ridge allele status. The DNA test now needs to be extended to a larger group of dogs, so as to confirm its reliability and effectiveness as a diagnostic test.

*Remember: each dog has one copy of each chromosome from each parent. So there are two copies of each chromosome and all the genes on them. However, because one set has come from each parent, they may not contain the same version of some genes on both chromosome pairs.

## Background

Modern genomic approaches have revealed the molecular basis for a variety of breed-specific traits and/or conditions. A particular copy number variation (CNV) plays an important role in the development of the breed-defining dorsal ridge in two related breeds, the Rhodesian Ridgeback and the Thai Ridgeback [1].

Specifically, duplication of a ~133,000 bp region on chromosome 18 has been identified in all dogs exhibiting a ridge; this duplication is absent in dogs without a ridge [1]. Previous work [2] indicated that the ridge trait is inherited in a dominant fashion; thus a single copy of this duplication results in appearance of a ridge, and dogs with two copies of the duplication are indistinguishable from dogs with one copy. We refer to this duplication as the Ridge allele.

Dogs with two copies of the Ridge allele are at substantially greater risk for the development of dermoid sinus [1], a neural tube defect that is caused by incomplete separation of tissue layers during development. Affected dogs develop an inappropriate connection between the dermal surface and the spinal cord. This growth can become infected and lead to the development of abscesses and sepsis. In many cases, dermoid sinus can be fatal. Of twelve dogs with dermoid sinus examined, ten were shown to have two copies of the Ridge allele [1]. There are no reports of dermoid sinus in ridgeless dogs [3].

Therefore, there are two selection pressures are being applied simultaneously to the Ridge allele by modern breeding practices. The ridge is important to breed identity (in fact, a ridgeless Rhodesian Ridgeback cannot be AKC registered), so there is no desire to remove dogs harboring this dominant mutation from the breeding pool. However, in some dogs, the Ridge allele will lead to the development of dermoid sinus, a malady that we would like to select against. In order to make truly informed decisions about how to breed these dogs—to minimize dermoid sinus and maximize ridge appearance—a way to discriminate between dogs with one and two copies of the Ridge allele would be useful.

In order to genotype Rhodesian Ridgeback dogs for the Ridge allele, conventional DNA sequencing is not sufficient; other methodologies must be employed. Multiplex Ligation dependent Genome Amplification (MLGA) has been used for Ridge allele genotyping [1]. While reportedly fast and accurate, this approach requires analysis of products by capillary electrophoresis and other analytical techniques that are not available in laboratories without certain types of DNA sequencing equipment. A variety of other methods have been used for CNV genotyping, ranging from conventional Southern blotting to next-generation high-throughput DNA sequencing. Frequently, a microarray or quantitative PCR approach is taken [4]. Because of our familiarity with the technique and its relatively low cost, we chose SYBR Green-based quantitative PCR as the basis for a Ridge allele genotyping test. As described below, this protocol is relatively easy to perform and reliably produces accurate genotype results for dogs of known genotype.

## Results

### Test design

**Figure 1:**
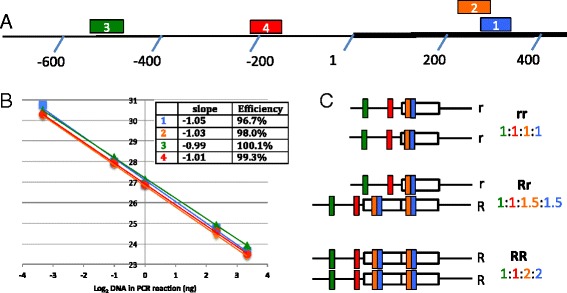
**Characterization of the primers used in the test. A**. The region of chromosome 18 under investigation is shown, with the repeat region indicated by a darker line, and chromosome position indicated as +/- with respect to the first nucleotide of the repeat. Four primer sets are used, and the regions amplified by each are indicated as 1, 2, 3 and 4 in the image. Primer sets 1 and 2 amplify a region within the repeat, primer sets 3 and 4 amplify a region outside of the repeat. **B**. Primer efficiency for each set of primers. Four different concentrations of starting template were amplified with each primer set and Ct values were recorded on the y-axis. Colors used are the same as in panel A. *Inset:* slopes from the best-fit lines and corresponding calculated efficiency values. **C**. Location of the regions amplified in dogs of three different genotypes. Colors used are the same as in panel A. The ratio of products expected for each genotype is indicated.

The design of our test is based in the ability of quantitative PCR to detect subtle differences in the amount of starting material in a PCR reaction. Compared to dogs with no ridge, those with one copy of the Ridge allele should have 50% more DNA representing the repeat region, while dogs with two copies of the Ridge allele should have 100% more. Therefore, we selected an area within the repeat region to quantify through PCR amplification. In order to normalize these values, an area just outside the repeat region was selected. To minimize the possibility of results stemming from experimental artifacts, two sets of primers representing both areas were selected (Figure 1A).

To characterize these primer pairs, a serial dilution of canine DNA was subjected to amplification (Figure 1B). In theory, each cycle of PCR should result in a doubling of the amount of target sequence; when this occurs, it is referred to as 100% efficiency. For all primer pairs, the amplification efficiency was near 100% when tested on dogs without the ridge allele (see inset, Figure 1B), or dogs harboring one or two copies of the duplication (not shown). This finding allowed us to ignore this variable in our genotyping analysis, since all primer sets amplified with similar efficiency [5]. In addition, analysis of melt curves of the products generated was consistent with the production of a single amplicon, with no evidence of primer-dimer formation (not shown).

### Test validation on dogs of known genotype

If a dog has been bred, one can, in some cases, infer the dog’s genotype with respect to the Ridge allele. For example, the genotypes of two ridged dogs that produce ridgeless puppies must be heterozygous (Rr), while a ridged dog with a large number of offspring with different mates, that produces no ridgeless puppies, is likely to be homozygous for the ridge allele (RR). DNA samples from a ridgeless dog of mixed breed, a ridgeless Rhodesian Ridgeback, a heterozygous Rhodesian Ridgeback and a likely homozygous Rhodesian Ridgeback were obtained and subjected to our quantitative PCR test. Four reactions, each utilizing a different primer set, were run in parallel for each dog. Following amplification, an arbitrary point of fluorescence accumulation was selected as the threshold (to represent early exponential accumulation in the majority of reactions), and each experiment used this threshold value to generate the corresponding Ct (threshold cycle) value (Table 1). Thus, the point at which exponential amplification occurs can be compared between experiments representing different dogs and primer sets.

**Table 1:** **Average Ct values for various dogs (SEM)**

	**1**	**2**	**3**	**4**
Ridgeless mixed breed dog (rr)	24.97 (0.04)	24.56 (0.08)	25.09 (0.05)	24.74 (0.02)
Ridgeless Rhodesian Ridgeback (rr)	25.76 (0.04)	25.67 (0.06)	26.16 (0.04)	25.88 (0.10)
Rhodesian Ridgeback with 1 Ridge allele (Rr)	25.15 (0.08)	24.92 (0.10)	25.87 (0.07)	25.70 (0.08)
Rhodesian Ridgeback with 2 Ridge alleles (RR)	23.92 (0.06)	23.55 (0.03)	25.05 (0.04)	24.81 (0.09)

The Ct values can be used to determine the relative amount of DNA in the starting material for each reaction. Two control aspects must be considered in this calculation. First, the amount of DNA in the repeated region is compared to the amount of DNA in the control region (outside of the repeat). This concept is illustrated in Figure 1C, where we see that a dog without a ridge would be expected to possess the same amount of DNA within and without the repeat region, while a Rr dog would have 50% more repeat region DNA and a RR dog would have 100% more repeat region DNA than the control region. A second point of comparison is used to correct for differences in the amplification products themselves. Here, we compare the test dogs (in this case three Rhodesian Ridgeback dogs of different genotypes) to the control mixed breed dog. This allows us to calculate Relative Copy Number (RCN); a value that is relative to the copy number found in the control mixed breed dog. Thus, for a test sample from a dog without a ridge, the relative copy number (RCN) should be 1, while a dog with a single copy of the Ridge allele would be expected to generate a RCN of 1.5 and a dog with two Ridge alleles would be expected to generate a 2.0.

**Figure 2:**
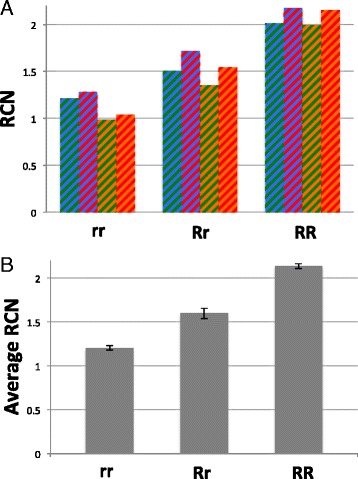
**Genotype determination. A**. Three dogs of known genotype were selected. DNA from these dogs was analyzed by qPCR and RCN values calculated. Each bar represents a different set of comparison of PCR products: Blue/Green refers products 1 and 3; Blue/Red is 1 and 4; Orange/Green is 2 and 3; Orange/Red is 2 and 4. **B**. Average RCN values were calculated for each dog and the experiment repeated four times so that values could be compared to each other. All three means were shown to be different from each other (p << 0.05).

For the three test subjects, RCN was computed by looking at all four possible combinations of primer pairs. The result (Figure 2A) is that dogs of different genotypes can be clearly discriminated from each other and that the values obtained are very close to the expected values. We calculated the mean of the RCN for each dog to generate Average RCN values: rr = 1.04; Rr = 1.53; RR = 2.17, for the data shown in Figure 2A.

This experiment was repeated four times, so that average CRN values could be compared to each other (Figure 2B). ANOVA revealed that the three means were statistically distinguishable (p << 0.05) and all pairwise combinations showed statistically significant differences (p << 0.05). Thus, our test provides unambiguous results for each of the genotypes tested.

### Utility of test for predicting genotype

With this background, we next explored the ability of our test to make correct calls regarding dog genotypes. DNA was extracted and analyzed as described above, and Average RCN values were calculated for five different test subjects. Test subjects were all Rhodesian Ridgeback dogs in which genotype could be inferred based on breeding or other history. These Average RCN values were compared to the three dogs described above. Paired t-tests were employed to either exclude or include an Average RCN as being statistically indistinguishable from the Average RCN for each genotype (Table 2). In all cases, a single genotype was consistent with the data, and this genotype was assigned to the test subject. In all cases, the test subject’s experimentally determined genotype was in agreement with its predicted genotype.

**Table 2:** **Genotype assignments for five test dogs**

**Average RCN (SEM)**	**Different than rr?**	**Different than Rr?**	**Different than RR?**	**Assigned genotype**	**Known info about dog**
1.63 (0.04)	yes	no	yes	**Rr**	Female, produced ridgeless puppies
2.23 (0.05)	yes	yes	no	**RR**	Female, has dermoid sinus, no offspring
1.65 (0.06)	yes	no	yes	**Rr**	Male, produced ridgeless puppies
0.99 (0.01)	no	yes	yes	**rr**	Female, ridgeless Rhodesian Ridgeback
2.25 (0.14)	yes	yes	no	**RR**	Male, produced only ridged puppies

## Discussion

For years, the genetic basis for the Ridge allele has been understood [1], but there has not been a validated methodology available for genotyping individual dogs. This work shows that a rapid and relatively inexpensive qPCR approach works to correctly call genotype.

Collecting saliva samples from dogs is straightforward and easily accomplished by most dog owners. Once a sample is received through the mail, DNA extraction and quantification takes less than three hours, and multiple samples can be processed simultaneously. Setting up the reactions, running the protocol and evaluating the results takes approximately 4 hours. Thus, results can be obtained within a very short time period. The primary expense associated with this protocol is the labor associated with processing the samples, the saliva collection kit, and qPCR reagents. All together, it is reasonable to expect that a well-equipped lab could provide this service at a price-point comparable to other canine genetic tests.

Genotyping of the Ridge allele in Rhodesian Ridgeback dogs addresses a fairly unique problem. Genetic transmission of the allele is desired, as the ridge trait is a hallmark of the breed. At the same time, the Ridge allele is associated with a severe health problem for some dogs. Without the ability to accurately determine a dog’s genotype with respect to the Ridge allele, breeders have had to make guesses about dogs when planning breedings. This type of approach can generate puppies that are undesirable, either as ridgeless dogs or as dogs with dermoid sinus. Both of these outcomes can be minimized if a dog’s Ridge genotype is known prior to breeding. For example, if a dog is determined to be homozygous for the Ridge allele, mating with a heterozygous dog (rather than another homozygote) should decrease the appearance of dermoid sinus in the offspring.

While surgery exists as an option for puppies with dermoid sinus, a far better solution would be to prevent this condition in the first place. Some preliminary work has looked at the role of folate supplementation before and during pregnancy in preventing dermoid sinus [6]. This connection is somewhat logical given the role that folate supplementation has been shown to play in preventing neural tube defects in humans [7]. However, this work did not explore the direct connection between genotype and dermoid sinus, so the role of folate as a preventative agent is as yet unsettled. Therefore, the basis for the ambiguous genetics associated with dermoid sinus may be due to environmental factors, additional as-yet unidentified genetic contributions, or a combination of these two.

This Ridge allele genotyping test provides a basis for exploring the environmental and/or genetic factors that contribute to the development of dermoid sinus in dogs homozygous for the Ridge allele. For example, a well-designed test can now be developed that will address the question of whether pre-natal folate supplementation has a role in preventing dermoid sinus. Being able to accurately genotype dogs in such a study will allow for an experimental effect to be more accurately detected. In addition, a genome wide association screen to detect genetic contributions to the development of dermoid sinus can now be designed that utilizes RR dogs with dermoid sinus as the cases and RR dogs without dermoid sinus as the controls.

## Conclusions

We have developed a test for accurately determining the genotype of Rhodesian Ridgeback dogs with respect to the copy number of the Ridge allele.

## Methods

### Dogs and DNA samples

All dogs used in this study were in the care of their owners. Breeding histories, when appropriate, were collected by interviewing the dogs’ owners. DNA samples were collected by sending dog owners Oragene Animal OA-400 (DNA Genotek, Canada) animal saliva collection kits and asking them to follow the manufacturer’s instructions for saliva collection and then send the kit back via mail. DNA was purified in our laboratory following the manufacturers instructions. Following purification, DNA was quantified via Nanodrop (ThermoScientific) and diluted in dH_2_0 to an appropriate working concentration. This protocol was reviewed by the SUNY New Paltz Animal Care and Use Committee, and owners provided consent for the procedures.

### Quantitative PCR

Reactions were generally designed in quadruplicate in a final volume of 10 μl and contained 1X iTaq SYBR Green Master Mix (BioRad), 400 nM forward and reverse primers and 2 ng genomic DNA. Amplification (Bio Rad CFX96) was initiated with a 5 minute incubation at 95°C, and then 40 cycles of 95°C for 5 sec followed by 60°C for 30 sec. Melt curve analysis was employed to ensure single product of the appropriate size was generated. Primers used were: Set 1: 5′ TGCCGCTCAGATGATCAAC3′ and 5′TCTGCTTTTCTCTGCTCCC3′, Set 2: 5′ATTGGCAGTGTCCGTGTGAG3′ and 5′AAGCCCCGCAGACAATGAAC3′, Set 3: 5′GCATCCACCTAAGCAATCTG3′ and 5′CCCTATTCTCTTCCACCCATC3′, Set 4: 5′GCTTCTGCTTTGATACCCTTC3′ and 5′GTTCTGCAACAGCATCTCC3′.

### Data analysis

To calculate primer efficiency, we employed the equation E = (2^(−1/slope)^/2) ∗ 100. All primer pairs ranged from 97–100% efficiency, thus eliminating the need to correct for primer efficiency in subsequent calculations.

Relative copy number (RCN) was calculated by a variation of the ddCt method [8]. In our case, RCN = (2^(Ct control dog:repeat region – Ct test dog:repeat region)/(2^(Ct control dog:control region – Ct test dog:control region), where contol dog refers to a mixed breed dog without a ridge, test dog refers to the dog being genotyped, repeat region refers to the region on chromosome 18 within the repeated region (amplified by primer set 1 or 2), and control region refers to the region on chromosome 18 just outside of the repeated region (amplified by primer sets 3 or 4). Since four primer sets were used, we could calculate this value by comparing primer set 1 to 3 and 4 and primer set 2 to 3 and 4. Thus, four RCN values could be determined in each experiment. These values were then averaged to generate an Average RCN value. Experiments were repeated at least three times so that SEM values could be determined and used in paired t-tests in order to substantiate genotype calls.
